# Neuromodulatory effect of transcranial direct current stimulation on cue reactivity and craving in young adults with internet gaming disorder: an event-related potential study

**DOI:** 10.3389/fpubh.2024.1494313

**Published:** 2025-01-14

**Authors:** Sung Nyun Kim, Jung-Seok Choi, Minkyung Park, So Young Yoo, Areum Choi, Ja Wook Koo, Ung Gu Kang

**Affiliations:** ^1^Department of Psychiatry, Seoul Medical Center, Seoul, Republic of Korea; ^2^Department of Psychiatry, Samsung Medical Center, Sungkyunkwan University School of Medicine, Seoul, Republic of Korea; ^3^Department of Psychiatry, SMG-SNU Boramae Medical Center, Seoul National University College of Medicine, Seoul, Republic of Korea; ^4^Emotion, Cognition and Behavior Research Group, Korea Brain Research Institute, Daegu, Republic of Korea; ^5^Department of Brain and Cognitive Sciences, Daegu Gyeongbuk Institute of Science and Technology, Daegu, Republic of Korea; ^6^Department of Psychiatry and Behavioral Science, Seoul National University College of Medicine, Seoul, Republic of Korea

**Keywords:** tDCS, ERP, cue reactivity, craving, internet gaming disorder

## Abstract

**Objective:**

This study assessed the effects of transcranial direct current stimulation (tDCS) on cue reactivity and craving for game-related cues using event-related potentials (ERPs) in internet gaming disorder (IGD) patients.

**Methods:**

At baseline, a series of game-related and neutral pictures were shown to both IGD and healthy controls (HCs) while ERPs were recorded. Late positive potentials (LPP) were used to investigate cue reactivity. During intervention, IGD patients received 10 sessions (two sessions/day for 5 consecutive days, 2 mA for 20 min/session) of tDCS to the left (anode stimulation) and right (cathode) dorsolateral prefrontal cortex. Subjectively assessed craving and LPP component was analyzed before stimulation and at the 1-month follow-up after tDCS in IGD.

**Results:**

At baseline, patients with IGD showed higher LPP amplitudes for game-related cues in the centro-parietal and parietal regions than HCs. After 10 sessions of tDCS, increased LPP amplitudes decreased significantly at 1-month follow-up., as well as subjective craving for gaming.

**Conclusion:**

These findings suggest that neurophysiological arousal in response to game-related cues in the IGD group could be modulated by the effects of tDCS. LPP was a significant neurophysiological marker of the neuroplastic response of cue reactivity underlying the therapeutic effect of tDCS on IGD. Based on the present findings, tDCS could be expanded to the treatment of other addictive disorders, including substance use disorder and behavioral addictions.

## 1 Introduction

Some users of internet games may experience negative outcomes in terms of academic or occupational performance, interpersonal relationships, or mental well-being (1). Internet gaming disorder (IGD) has been conceptualized as a behavioral addiction because it shares core symptoms and neurobiological features with substance use disorder, including loss of control and craving (2). In 2013, IGD was included in the DSM-5 as a condition warranting further research (3). The World Health Organization has included “Hazardous gaming” and “Gaming disorder” in its 11th revision of the International Classification of Diseases (4). IGD manifests with notable prevalence worldwide, as recent meta-analyses estimated that the global prevalence of IGD is 1.96–2.47% (5, 6). While various treatments strategies have been employed for IGD (7), here remains the need for the development of better treatment based on the neurobiological mechanisms underlying IGD (8).

Cue reactivity and attentional bias have long been thought to play an important role in addictive behavior (9). Incentive-sensitization models of addiction assert that previously neutral stimuli become strongly associated with the reinforcing properties of an addictive drug during the development of dependence (10). Hence, these stimuli become excessively salient and acquire the potential to evoke addiction-like responses (10, 11). Incentive-sensitization models also assume that cues both induce craving and enhance attention toward drug cues, causing individuals to continue to relapse with respect to addictive drugs or behaviors (9, 12). Similar to substance addiction, attentional bias has also been identified in IGD and reported to be associated with craving (13, 14). Heuer et al. (13) demonstrated that individuals with IGD exhibit impairments in attention disengagement from computer-related stimuli. Similarly, Zhou et al. (14) found that attentional disengagement bias was associated with a greater increase in game craving immediately after encountering game-related stimuli.

The late positive potential (LPP) is often used in event-related potential (ERP) studies to represent attentional bias, which is strongly evoked by emotionally salient stimuli (15). LPP is an ERP that follows emotional stimuli by 350–700 ms (12), and an increase in the LPP amplitude to substance-related cues has been reported in various substance use disorders (16–18). Additionally, a decrease in LPP amplitude has been reported after treatment for alcohol use disorder (16). In line with findings in substance use disorders, subjects with IGD showed enhanced LPP amplitude in response to game-related cues compared to healthy controls in association with emotional arousal (15, 19). This suggests that LPP can be used as a marker of neurophysiological mechanism associated with cue reactivity in IGD, Therefore, examining the LPP as a marker of cue reactivity will allow us to test the hypothesis that neuromodulation reduces conditioned game-associated responses.

Transcranial direct current stimulation (tDCS) is a non-invasive brain stimulation technique in which a low-intensity direct current is applied over the scalp to modulate cortical excitability and induce neuroplasticity (20, 21). The safety profile of tDCS is very good; no serious adverse events or irreversible injuries have been reported (22, 23). Previous tDCS studies mostly targeted the dorsolateral prefrontal cortex (DLPFC) due to its association with cue-elicited craving and cognitive functions related to addiction, i.e., decision-making, inhibitory control, and attentional bias (24). A recent meta-analysis of 20 randomized controlled clinical trials on food, nicotine, alcohol, and drug abuse reported that neuromodulation with tDCS or repetitive transcranial magnetic stimulation (rTMS) in patients with these addictions led to a sustained decrease in cravings or substance use (25). A few studies have demonstrated that repeated tDCS on the DLPFC may affect IGD symptoms and improve emotion regulation (26) and craving (27). Moreover, a recent study showed that even a single session of tDCS enhanced inhibitory control in patients with IGD (28). However, no studies have directly investigated the neurophysiological effects of tDCS on the functioning of the cue-reactivity system in IGD patients. We assumed that if tDCS effects would be mediated through changes in attentional bias, its therapeutic impact could be sustained over an extended period. While previous studies have primarily focused on immediate or short-term effects (within 1 week post-tDCS), this study evaluated the intervention’s efficacy at a 1-month follow-up.

Therefore, in this study, we assessed the neurophysiological effects of repetitive tDCS on cue reactivity and craving for game-related cues using ERPs in patients with IGD. We specifically targeted the left DLPFC due to its crucial role in regulating emotional bias (29) and its significant association with craving observed in previous studies (30). We hypothesized that tDCS would decrease LPP associated with game-related cues, which would be higher at baseline compared with healthy controls (HCs). We also hypothesized that repetitive tDCS in IGD subjects would have effects on clinical measures such as symptom severity, impulsivity, and subjective craving.

## 2 Materials and methods

### 2.1 Participants

We recruited 32 young adult males (12 patients with IGD and 20 HCs) from SMG-SNU Boramae Medical Center in Seoul, South Korea. All of the IGD patients were seeking treatment for problems related to excessive internet gaming. IGD was diagnosed according to DSM-5 criteria. We exclusively recruited male participants due to the higher prevalence of IGD in males and to minimize the influence of sex-related neurobiological differences (6). The participants had no comorbid psychiatric diagnoses, including attention deficit hyperactivity disorder (ADHD) and depressive or anxiety disorders, had had no history of head injury or cognitive delay.

All participants were medication-naive at the time of the assessment and during the tDCS intervention. The Korean version of the Wechsler Adult Intelligence Scale-IV (WAIS-IV) was administered to all subjects to estimate the intelligence quotient, and only subjects with WAIS-IV scores >80 were included in the study.

### 2.2 Clinical assessments

Young’s Internet Addiction test [IAT]: IGD severity was assessed using Young’s IAT, which includes 20 items rated using a 5-point scale with total scores ranging from 20 to 100 (31–33).

Barratt Impulsiveness Scale-11 [BIS-11]: The BIS-11 assesses impulsivity based on three subscales of cognitive impulsiveness (e.g., “I get easily bored when solving thought problems”), motor impulsiveness (e.g., “I do things without thinking”), and non-planning impulsiveness (e.g., “I am more interested in the present than in the future”) (34).

Craving for gaming: the participants were asked to rate their craving in response to the game-related cues using a visual analog scale (VAS) ranging from 0(none) to 10 (maximum). VAS is widely acknowledged for its simplicity and effectiveness in quantifying subjective craving intensity, as demonstrated in numerous previous studies on substance addiction and IGD (19, 35–37).

### 2.3 Procedures

The research was fully explained, and all participants provided written informed consent before participating in the study. The participants received a monetary reward of about $50 USD for participation.

Baseline assessments included IGD severity (IAT), psychological measures of impulsivity (BIS-11), ERPs for both groups, and craving for internet gaming in IGD. During intervention, patients with IGD underwent 10 sessions (2 sessions per day for 5 consecutive days) of tDCS. After repeated tDCS, the ERPs, clinical status (IAT and BIS-11), and subjective craving for gaming were assessed at 1-month follow-up to investigate the short-term effects of tDCS rather than the immediate effect.

This study was conducted following the Declaration of Helsinki and was approved by the Institutional Review Board of SMG-SNU Boramae Medical Center, Seoul, Republic of Korea (IRB No. 30–2017-10).

### 2.4 Intervention: tDCS

The tDCS anode was placed over the left DLPFC (F3), and the cathode was placed over the right DLPFC (F4) according to the 10–20 international system. The current flowed continuously during the two 20-min stimulation periods (2.0 mA) separated by a 20-min rest interval (no stimulation; a 20:20:20 schedule). This protocol was based on that of previous studies (38, 39). After the baseline visit, the participants received 10 active sessions (two sessions per day for 5 consecutive days) using the tDCS device (Ybrain, Seongnam, South Korea). The participants were asked to report any adverse effects after each session. All 12 patients with IGD successfully completed the repetitive tDCS but 2 patients were excluded during EEG data cleaning. Finally, 10 patients with IGD were included in the analysis to assess the effects of tDCS.

### 2.5 EEG and clinical measurements

#### 2.5.1 EEG recording and cue reactivity task

We used the electrophysiological marker LPP to compare cue reactivity and craving between patients with IGD and HC at baseline, and before and after the tDCS intervention in patients with IGD. The LPP component was measured before the tDCS intervention and at the 1-month follow-up visit. The LPP was evoked through a cue reactivity task. The cue reactivity task involved two picture sets: in one set, game-related cues were included in screen captures of three popular internet games (League of Legends, FIFA, and Sudden Attack); in the other set, neutral pictures were taken from the neutral category of the International Affective Picture System (58). Each individual visual stimulus was matched for size (resolution of 1,024 × 768 pixels, 361 mm × 271 mm, 72 dpi), luminance, brightness, and color. Each category (League of Legends, FIFA, Sudden Attack, and Neutral) consisted of seven different pictures, and a pseudo-random series of pictures was repeated six times during the task. The stimulus duration was 3,000 ms, and the inter-stimulus interval was 2,000 ms.

The participants were seated comfortably in a dimly lit, electrically shielded room and instructed to watch the screen carefully. Continuous EEG was recorded using a Neuroscan 64-channel SynAmps system with a 64-channel Quick-Cap based on the modified 10–20 international system (Compumedics, Charlotte, NC, United States). Electrodes at the mastoid sites served as reference electrodes, and the ground electrode was placed between the FPz and Fz electrode sites. The EEG was digitized at a 1,000-Hz sampling rate with an online 0.05–100 Hz filter. Eye movement artifacts were monitored by recording vertical and horizontal electrooculograms using electrodes below and on the outer canthus of the left eye. The resistance at all electrode sites was <5 kΩ.

#### 2.5.2 ERP analysis

The ERPs data were pre-processed using Curry 7.0 software (Compumedics). The EEG recordings were adjusted to a common average reference, and eye movement artifacts were reduced using the artifact reduction algorithm in Curry software (59). The continuous EEG data were bandpass filtered between 0.1 and 30 Hz, epoched to a 200-ms pre-stimulus and 3,000 ms post-stimulus, and baseline-corrected using the average pre-stimulus interval voltage. Epochs containing EEG amplitudes that exceeded ±100 mV were rejected automatically. The epochs were averaged separately for each class (Game vs. Neutral). The late positive potential (LPP) is enhanced for emotionally salient stimuli compared to control stimuli between 350 and 700 ms poststimulus onset (40, 41). LPP were calculated as the mean values of amplitudes between 350 and 700 ms at the parietal (P3, P1, Pz, P2, and P4) and the centroparietal (CP3, CP1, CPz, CP2, and CP4) electrode sites. The electrode sites were selected as reported previously (15).

### 2.6 Statistical analysis

Demographic and clinical variables were compared between the patients with IGD and the HCs using Student’s *t*-test or Welch’s *t-*test after testing for homogeneity the variance. Clinical data were compared before and after the tDCS intervention using the paired *t*-test or Wilcoxon signed-rank test after performing the Shapiro–Wilk normality test. To examine the association between changes in clinical data (IAT, BIS, and craving) and changes in LPP amplitude, Spearman’s rank correlation analysis was performed. The neuromodulatory effects of tDCS on mean LPP amplitude were analyzed using repeated-measures analysis of variance (rmANOVA) with the electrode site (P3, P1, Pz, P2, P4, CP3, CP1, CPz, CP2, and CP4,) and condition (Game and Neutral) as within-subject factors, and group (IGD, HC) as the between-subjects factor. Group comparisons of the mean LPP amplitudes were performed using rmANOVA with the 10 electrode sites as within-subject factors and group (HC and IGD) as the between-subjects factor. *p*-values were Greenhouse–Geisser corrected where appropriate. The paired *t*-test was used to compare the difference in mean LPP amplitudes before and after the tDCS intervention at electrode sites showing significant group differences at baseline. SPSS software (version 25.0; IBM Corp., Armonk, NY, United States) was used for statistical analyses. A *p*-value <0.05 was considered significant.

## 3 Results

### 3.1 Demographic and clinical data

No significant group difference in age was observed with mean age 25.45 (SD = 2.83, HCs) vs. 23.00 (SD = 6.79, IGD). The clinical data are shown in Table 1. Y-IAT (*t*[30] = −6.73, *p <* 0.001) and BIS-11 (*t*[30] = −5.16, *p <* 0.001) scores exhibited significant group differences.

**Table 1:** Demographic and clinical characteristics at baseline (HCs and IGD) and at 1-month follow-up in IGD.

	Healthy control (*n =* 20)	Internet gaming disorder (*n =* 12)	*t*	*P*
Baseline assessments				
IAT	34.10 (9.14)	59.25 (11.91)	−6.726	<0.0001
BIS-11	54.15 (6.89)	68.33 (8.53)	−5.155	<0.0001

	Baseline	1-month follow-up	*z/t*	*P*
Between tDCS interventions in IGD (*n* = 10)				
IAT	61.70 (11.51)	54.10 (16.20)	−1.378	0.168
BIS-11	67.70 (8.59)	67.20 (7.18)	0.188	0.855
Craving (VAS)	6.80 (1.32)	5.10 (2.02)	−2.050	0.040

IAT, Young’s Internet addiction test; BIS-11, Barratt impulsiveness scale-11; tDCS, transcranial direct current stimulation; VAS, Visual Analog Scale.

Craving, as measured by the VAS, decreased significantly after the tDCS intervention [z = −2.05, *p =* 0.04] in subjects with IGD. However, the IAT and BIS-11 scores did not differ significantly before and after the tDCS intervention.

### 3.2 LPP amplitudes

At baseline, the game-related cues elicited significantly higher LPP amplitudes in patients with IGD than in HCs at the CP3, CP1, P3, P1 and Pz electrode sites (Table 2). A significant main effect of condition (game and neutral) was observed on mean LPP amplitude (*F*[1, 30] = 10.531, *p =* 0.003). A significant group effect (*F*[1, 30] = 2.921, *p =* 0.034] and a group by electrode site interaction (*F*[2.560, 30) = 3.611, *p =* 0.022) were detected on the mean LPP amplitude elicited by game-related pictures. No significant group effect was detected in the neutral condition (*F*[1, 30] = 2.828, *p =* 0.103). After the tDCS intervention, patients with IGD had decreased LPP amplitudes at the CP1, P3, P1, and Pz electrode sites for the game-related conditions compared to baseline (Table 3). No significant correlation was found between changes in clinical data (such as craving) and changes in LPPs after correcting for the *p*-value.

**Table 2:** Comparison of late positive potentials (LPPs) that averaged between 350 and 700 ms post-stimulus onset across the IGD and HC groups.

	Healthy control (*n =* 20)	Internet gaming disorder (*n* = 12)	*T*	*p*
	Mean (SD)	Mean (SD)		
Game stimuli				
CP3 electrode site	0.85 (0.85)	1.81 (0.88)	−3.053	0.005^*^
CP1 electrode site	0.87 (0.77)	1.90 (1.20)	−2.959	0.006^*^
CPz electrode site	0.47 (1.17)	1.03 (1.06)	−1.525	0.138
CP2 electrode site	0.88 (0.89)	1.19 (1.37)	−0.784	0.439
CP4 electrode site	1.87 (1.02)	1.27 (0.84)	1.712	0.097
P3 electrode site	3.39 (1.76)	5.12 (2.93)	−2.090	0.045^*^
P1 electrode site	2.95 (1.54)	5.11 (3.28)	−2.546	0.016^*^
Pz electrode site	2.47 (1.30)	4.21 (2.83)	−2.369	0.024^*^
P2 electrode site	2.82 (1.13)	4.38 (3.15)	−2.025	0.052
P4 electrode site	3.99 (1.55)	4.93 (3.03)	−1.175	0.249
Neutral				
CP3 electrode site	0.56 (0.84)	1.25 (0.81)	−2.284	0.030^*^
CP1 electrode site	0.43 (1.16)	1.05 (0.76)	−1.631	0.113
CPz electrode site	0.36 (1.28)	0.62 (0.98)	−0.610	0.547
CP2 electrode site	0.74 (1.32)	0.65 (0.98)	0.599	0.554
CP4 electrode site	1.55 (1.37)	0.44 (0.94)	1.995	0.055
P3 electrode site	2.98 (1.68)	4.71 (2.63)	−2.281	0.030^*^
P1 electrode site	2.64 (1.81)	4.14 (2.70)	−1.883	0.069
Pz electrode site	1.90 (1.65)	3.52 (2.68)	−2.134	0.041^*^
P2 electrode site	2.40 (1.71)	4.15 (3.07)	−2.076	0.047^*^
P4 electrode site	3.63 (2.02)	4.71 (3.24)	−1.166	0.253

* indicates *p* < 0.05.

**Table 3:** Changes of late positive potential after tDCS intervention in patients with IGD.

	Before (*n =* 10)	After (*n* = 10)	*T*	*p*
	Mean (SD)	Mean (SD)		
Game stimuli				
CP3 electrode site	1.84 (0.90)	1.02 (0.97)	1.625	0.139
CP1 electrode site	1.70 (0.97)	0.59 (1.01)	2.871	0.018^*^
P3 electrode site	4.39 (1.33)	2.68 (1.45)	3.022	0.014^*^
P1 electrode site	4.30 (2.12)	2.24 (1.65)	2.749	0.023^*^
Pz electrode site	3.57 (2.01)	1.85 (1.87)	2.693	0.025^*^
Neutral				
CP3 electrode site	1.30 (0.86)	0.77 (0.99)	1.123	0.291
P3 electrode site	4.30 (1.69)	2.04 (1.03)	3.413	0.008^*^
Pz electrode site	3.12 (2.01)	0.80 (1.64)	3.165	0.011^*^
P2 electrode site	3.49 (1.66)	1.62 (1.69)	2.730	0.023^*^

* indicates *p* < 0.05.

**Figure 1:**
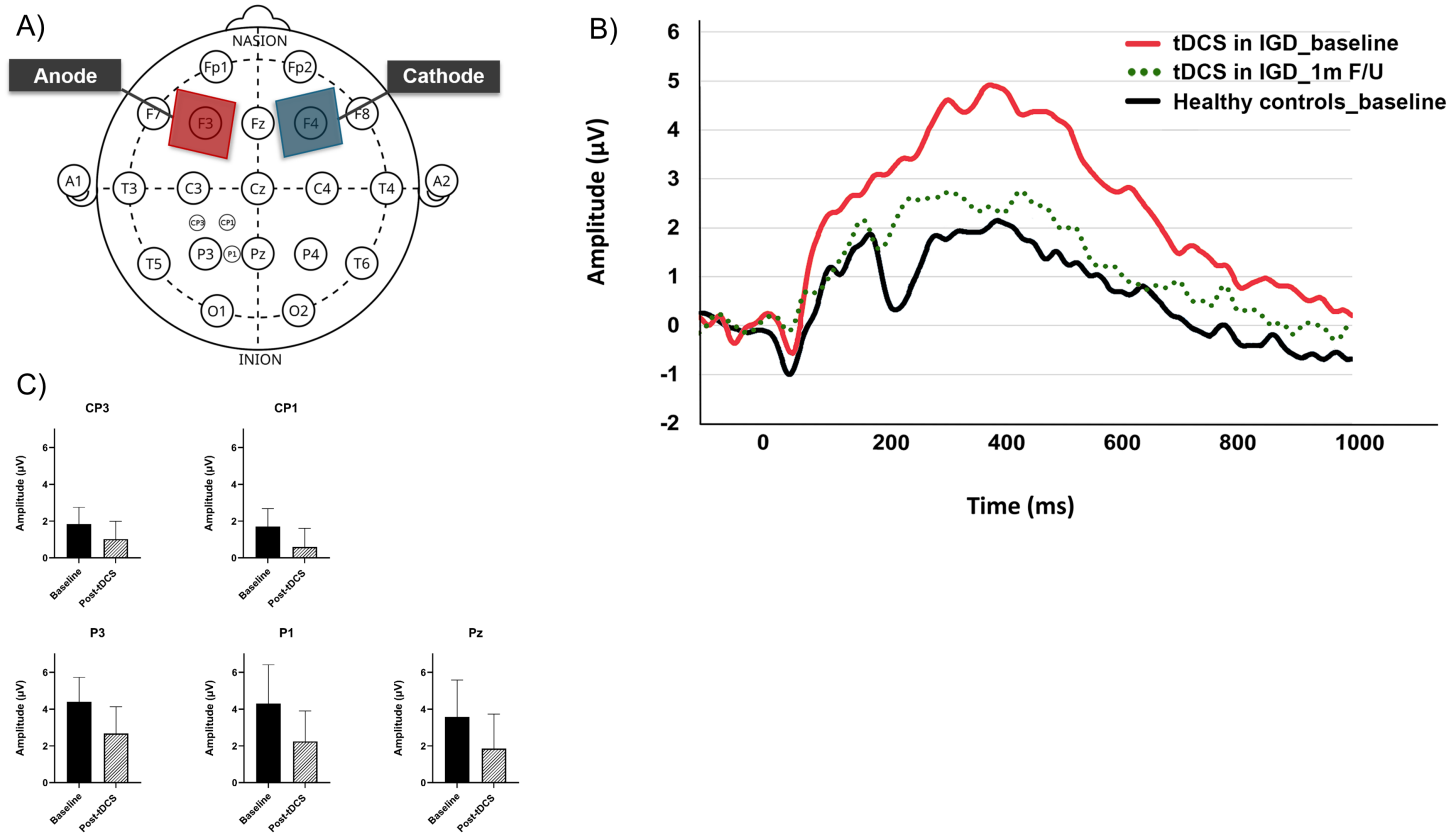
**(A)** tDCS electrode placement and ERP electrode sites including CP3, CP1, P3, P1, and Pz. **(B)** Changes of late positive potential (baseline and post-tDCS at 1 month f/u) at electrodes (CP3, CP1, P3, P1, and Pz). **(C)** Late positive potential waveforms elicited by the game-related stimuli at the P3 electrode site (baseline, post-tDCS at 1 month f/u).

Figure 1 displays (A) tDCS electrode placement, (B) LPP amplitude changes at CP3, CP1, P3, P1 and Pz and (C) the LPP waveforms elicited by the game-related stimuli in the IGD group at the P3 electrode site (baseline, post-tDCS at 1 month f/u).

## 4 Discussion

To the best of our knowledge, this is the first study to assess the neurophysiological effects of tDCS in subjects with IGD by analyzing ERPs during a cue-reactivity task. At baseline, this study observed increased cue reactivity to gaming stimuli in the IGD group, as measured by the LPP amplitude, compared to HCs as in line with previous study (19). Repetitive tDCS intervention demonstrated significant reduction in LPP amplitude that was previously increased by exposure to gaming stimuli in IGD patients after 1-month. Additionally, the tDCS treatment decreased subjective measures of craving for the game. However, in contrast to the hypothesis, tDCS did not show significant effects on IGD symptom severity or impulsivity in this study. No significant correlation was found between changes in clinical data (such as craving) and changes in LPPs.

The LPP amplitude toward game-related cues decreased after repetitive tDCS in patients with IGD at the most electrodes sites (CP1, P3, P1, and Pz) out of electrodes (CP3, CP1, P3, P1, and Pz) where baseline LPP amplitude increased in the IGD group compared to the normal control. LPP amplitude increase in IGD by game-related cues at baseline is in line with a previous study (19). Previous studies showed that LPP is related to emotional arousal in association with addiction-related stimuli. LPP have been proposed to reflect motivated attention across different substance use disorders (12). LPP changes were associated with treatment effect on neurocognitive or clinical characteristics such as emotional arousal, craving, or predicted relapse in substance addiction (12, 16, 42, 43). Another study suggested that direct stimulation of the prefrontal cortex reduces the LPP, implying that the prefrontal cortex may directly modulate activity in parietal attentional networks involved in the automatic processing of emotionally salient stimuli (44). In this study, differences were noted in craving, an important clinical aspect of addiction. Previous study demonstrated that attentional bias in IGD was significantly associated with increased craving following exposure to game-related events, highlighting the interplay between cue reactivity, attentional bias, and craving in this disorder (14). However, no significant differences were observed in the symptom severity of IGD after tDCS. The lack of differences in IAT symptom severity contradicts our hypothesis and may be attributed to the small sample size and sustained period of one-month follow-up to miss symptom changes after tDCS. Taken together, our results indicate that LPP can serve as a significant neurophysiological marker of cue reactivity and possibly reflect neurobiological change underlying treatment effect in patients with IGD.

There is evidence of a relationship between neuroplasticity and serotonin (5-hydroxytryptamine) (45). Neuroplasticity mechanisms are related to the efficacy of synaptic connections. Synapses can strengthen or weaken their efficacy via long-term potentiation or long-term depression, respectively. tDCS can affect synaptic neuromodulator concentration, and tDCS and serotonin enhance each other’s function (46). Interestingly, the serotonergic system may modulate cue reactivity and attentional bias in addiction (47). A previous study reported that emotional bias modification weakens game-related compulsivity (48). Given the established link between serotonin and both emotional bias and compulsivity (49), the modulation of serotonin function may play a pivotal role in IGD. The neuromodulatory changes observed in this study, as indicated by alterations in LPP amplitude, suggest that combining tDCS with serotonin function modulators could potentially enhance the effectiveness of craving control and relapse prevention strategies in the treatment of IGD.

Previous tDCS studies on addiction have targeted the DLPFC due to its association with cue-elicited craving and cognitive functions related to addiction, such as decision-making, inhibitory control, and attentional bias (24). Dysfunctional self-control and response inhibition in addiction are associated with reduced executive control and salience network activity (50). Brain imaging studies of addiction have observed a pattern of increased activity of the executive-control network with activation, including the DLPFC (51–55). Studies on subjects with IGD have also reported abnormalities in functional connectivity of brain networks related to cognitive control, executive function, motivation, and reward, all of which are associated with the DLPFC (54, 56, 57). Ma et al. (56) identified altered cue-reactivity brain regions in individuals with IGD using functional brain network analysis and found these abnormalities to be consistent with those reported in substance-related addictions. These findings emphasize the importance of cue reactivity as a central mechanism in IGD, as well as in substance addiction. Consequently, our results indicate that the DLPFC may represent a critical target for interventions designed to address the cognitive dysfunctions inherent in addiction.

Several studies have reported therapeutic effects of tDCS on IGD; however, there is considerable variability regarding the optimal tDCS parameters, such as electrode location and the duration of stimulation. A recent review examining the impact of tDCS on attentional bias suggested that the left DLPFC plays a crucial role in regulating emotional bias (29). Similar to our study, Jeong et al. (27) applied tDCS during 12 sessions with anodal left/cathodal right stimulation and reported improvements in self-control and inhibition based on self-report scales. It was proposed that tDCS electrodes have opposing effects on target surface brain areas, enhancing (anode) or reducing (cathode) cortical excitability (26). However, Wu et al. (28) applied repeated anode right/cathodal left stimulation to the DLPFC in patients with IGD and reported enhanced regulation of craving and emotional control through cognitive tasks. Moreover, Wu et al. (28) reported the effectiveness of a single session of tDCS for reducing IGD symptom severity through anode right/cathodal left stimulation of the DLPFC, although they did not observe a reduction in craving, which differed from our findings. The heterogeneity in the modulatory effects of tDCS on IGD features may be attributed to variations in the montage set or tDCS stimulation intensity. Further investigation of tDCS for treating IGD is necessary.

Our study has several limitations. First, our sample size was relatively small and included only male participants, which limits the generalizability of our findings. While similar sample sizes have been employed in prior IGD tDCS research [e.g., (27)], future research should aim to recruit larger samples to confirm and extend our findings. Second, although comorbid psychiatric conditions, such as ADHD and depressive disorders, are common in patients with IGD, we excluded patients with comorbidities; thus, our sample may not be fully representative of this population. Future studies are needed to investigate the associations between tDCS-induced neuromodulatory changes and psychiatric comorbid conditions in patients with IGD. Third, a 1-month follow-up may be too short to adequately assess the clinical effects of tDCS including changes in the severity of addiction and long-term neuromodulating effects. Fourth, LPP amplitude increased even in response to neutral stimuli in the patient group compared to the HC group before and after tDCS, suggesting that patients with IGD may have heightened sensitivity to visual stimuli in general. Further research is necessary to explore this aspect in more detail. Lastly, the effect of active tDCS was not compared to sham stimulation in this study. Despite these limitations, our findings provide insight into the neuromodulating effects of tDCS on cue reactivity and craving in IGD. Based on the present findings, tDCS could be expanded to the treatment of other addictive disorders, including substance use disorder and behavioral addictions.

## 5 Conclusion

This study demonstrated that repetitive tDCS application induced a significant reduction of previously increased LPP amplitude in response to gaming stimuli in IGD patients. Our results indicated that LPP may serve as a significant neurophysiological marker of cue reactivity, where the neuroplastic response to tDCS could be targeted during the treatment of patients with IGD. Further studies are necessary to investigate the long-term effects of tDCS on clinical symptoms and neurocognition in patients with IGD.

## Data Availability

The raw data supporting the conclusions of this article will be made available by the authors, without undue reservation.
